# ^18^F‐FDG PET/CT Reveals Characteristic Prefrontal‐Posterior Cingulate Hypometabolism in anti‐AMPAR Encephalitis to Understand Cerebral Function: Insights From an Eight‐Patient Cohort

**DOI:** 10.1111/cns.70633

**Published:** 2025-10-28

**Authors:** Hengri Cong, Leilei yuan, Pei Zheng, Xuan Xu, Wenyue Dong, DeCai Tian, Linlin Yin, Yueta Ma, Tian Song, Yanxue Zhao, Yun Xu, Mengting Zhang, Baitao Liu, Guoqiang Chang, Ke Sun, Wenping Ma, Lin Ai, Wangshu Xu

**Affiliations:** ^1^ Department of Neurology, Beijing Tiantan Hospital Capital Medical University Beijing China; ^2^ China National Clinical Research Center for Neurological Diseases, Beijing Tiantan Hospital Capital Medical University Beijing China; ^3^ Department of Nuclear Medicine, Beijing Tiantan Hospital Capital Medical University Beijing China; ^4^ School of Basic Medical Sciences Peking University Beijing China; ^5^ Peking University Aerospace School of Clinical Medicine Beijing China; ^6^ Department of Neurology Tianjin Medical University General Hospital Tianjin China; ^7^ Functional Neurosurgery Department, Beijing Children's Hospital Capital Medical University, National Center for Children's Health Beijing China; ^8^ Department of Neurosurgery, Beijing Children's Hospital, Capital Medical University, National Center for Children's Health Beijing China

**Keywords:** ^18^F‐FDG PET/CT, anti‐AMPAR encephalitis, cerebral function, metabolic patterns

## Abstract

**Purpose:**

Anti‐AMPAR encephalitis is a rare antibody‐mediated disorder, and its clinicoradiological profile is incompletely defined. We aimed to characterize the clinical presentation and cerebral metabolic patterns of Chinese adults with anti‐AMPAR encephalitis using ^18^F‐FDG PET/CT.

**Methods:**

From August 2016 to August 2022, we retrospectively identified patients from two neurology centers who were positive for anti‐AMPAR antibodies in serum and/or cerebrospinal fluid. Demographic data, presenting symptoms, MRI and ^18^F‐FDG PET/CT findings (Discovery Elite, GE Healthcare), treatment regimens, and outcomes were extracted and analyzed.

**Results:**

Eight patients (four women) were included; median age was 53 years (range 37–63). Anti‐AMPA2R antibodies were detected in seven cases and anti‐AMPA1R antibodies in one. Limbic manifestations dominated, particularly cognitive decline and abnormal behavior; severe disease necessitated intensive care support in one patient, who nonetheless achieved a good outcome. Malignancy was documented in three individuals (lung carcinoma, hepatocellular carcinoma, and thymoma), and three harbored additional neural autoantibodies. MRI was abnormal in five patients (62.5%). All five subjects who underwent ^18^F‐FDG PET/CT displayed prefrontal hypometabolism; three also showed posterior cingulate hypometabolism. Focal hypermetabolism was observed in the right temporal lobe (one case) and in the left thalamus (one case).

**Conclusion:**

Anti‐AMPAR encephalitis usually presents as limbic encephalitis and is marked by prefrontal and posterior cingulate hypometabolism on ^18^F‐FDG PET/CT. In some instances, hypermetabolism was observed in the basal ganglia or temporal lobes, possibly reflecting the local blood flow.

## Graphical abstract

Anti‐AMPAR encephalitis is an extremely rare form of antibody‐mediated encephalitis. Understanding the brain tissue hemodynamics is crucial for assessing the disease progression and guiding treatment. The metabolic status of brain tissue can serve as an objective indicator of the hemodynamics in extracranial blood vessels via ^18^F‐FDG PET/CT imaging.

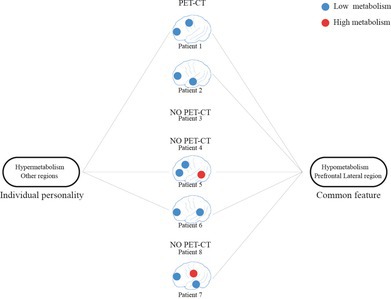

**Abbreviations**
^18^F‐FDG PET/CT: 18fluorine‐fluorodeoxyglucose‐positron emission tomography computed tomography scanner
AE: autoimmune encephalitis
AMPA: anti‐alpha‐amino‐3‐hydroxy‐5‐methyl‐4‐isoxazole propionic acid
AMPAR: anti‐alpha‐amino‐3‐hydroxy‐5‐methyl‐4‐isoxazole propionic acid receptor
ANA: antinuclear antibody
CASPR2: contactin‐associated protein‐like 2
CBA: cell‐based assays
COVID‐19: coronavirus disease 2019
CRMP5: collapsin response mediator protein 5
CSF: cerebrospinal fluid
EEG: electroencephalogram
GABAB‐R: γ‐aminobutyric acid‐B receptor
GAD65: glutamic acid decarboxylase 65‐kilodalton isoform
ICU: intensive care unit
IVIG: intravenous immunoglobulin
IVMG: intravenous methylprednisolone
IVMP: intravenous methylprednisolone
LE: limbic encephalitides
LGI1: leucine‐rich glioma‐inactivated protein 1
mEPSCs: miniature excitatory postsynaptic currents
MMF: mycophenolate mofetil
MRI: magnetic resonance signal
NMDAR: N‐methyl‐D‐aspartate receptor
PLEX: plasma exchange
PNMA: paraneoplastic Ma family

## 1 Introduction

Anti‐AMPAR encephalitis is a rare autoimmune disorder in which antibodies target the neuronal surface α‐amino‐3‐hydroxy‐5‐methyl‐4‐isoxazolepropionic acid receptor (AMPAR), producing a limbic‐encephalitis syndrome of short‐term memory loss, seizures, and psychiatric disturbance [1]. Most reported cases are sporadic, though atypical clinical manifestations have been increasingly documented in recent years [2, 3, 4].

Although extracranial vascular hemodynamic monitoring is now feasible, technical barriers to direct intracranial flow measurement have limited insights into the hemodynamics of anti‐AMPAR encephalitis. Consequently, it remains unclear whether extracranial hemodynamic data faithfully reflect cerebral function as captured by AMPAR positron emission tomography (PET) metabolic imaging.

AMPARs are ionotropic glutamate receptors assembled from four principal subunits, GluA1–4, together with various auxiliary subunits [5]. They are widely expressed throughout the central nervous system, with GluA1 and GluA2 especially abundant in the hippocampus and other limbic structures. PET is highly sensitive in autoimmune encephalitis, and distinct antibody subtypes yield characteristic metabolic signatures [6]. ^18^F‐FDG PET/CT, for example, discloses characteristic patterns in Leucine‐rich glioma‐inactivated 1 (LGI1)‐ and N‐methyl‐D‐aspartate receptor (NMDAR)‐mediated encephalitis. In anti‐NMDAR encephalitis, patients typically show frontal hypometabolism with medial temporal hypermetabolism, producing the well‐known “frontotemporal dissociation” pattern; reduced metabolism in the mesial occipital cortex is another hallmark of this entity [7]. In anti‐LGI1 encephalitis, unilateral or bilateral medial temporal hypermetabolism, most pronounced in the hippocampus, often coexists with basal ganglia metabolic abnormalities. This hypermetabolic profile is viewed as characteristic of anti‐LGI1 encephalitis and reflects active inflammation [8]. Notably, such biphasic metabolic changes are not confined to anti‐NMDAR disease but also appear in other antibody‐mediated encephalitides. Accordingly, ^18^F‐FDG PET/CT metabolic patterns aid early recognition of disease and help gauge its severity.

Against this backdrop, we aimed to investigate whether patients with different anti‐AMPAR antibody subtypes exhibit distinct cerebral metabolic patterns on ^18^F‐FDG PET/CT in this study, and analyzed ^18^F‐FDG PET/CT metabolic patterns, treatment courses, and outcomes in a cohort of Chinese patients with anti‐AMPAR encephalitis.

## 2 Methods

### 2.1 Cohort Ascertainment

Data were collected retrospectively from August 2016 to August 2022 at the Departments of Neurology of Beijing Tiantan Hospital, Capital Medical University, and Tianjin Medical University General Hospital. Among 320 admissions for autoimmune encephalitis, eight patients (four females, four males) fulfilled diagnostic criteria for anti‐AMPAR encephalitis, seven with anti‐AMPA2 receptor antibodies and one with anti‐AMPA1 receptor antibodies. Detailed clinical information for all patients was gathered from the patients themselves, their family members, or clinical records.

### 2.2 Inclusion Criteria and Data Collection

Eligible cases required a positive anti‐AMPA receptor autoantibody result in serum or cerebrospinal fluid (CSF) against neuronal surface antigens. Demographic characteristics, presenting symptoms, disease course, treatment regimens and responses, outcomes, and ancillary test results (serum and CSF analyses, brain magnetic resonance imaging (MRI), scalp electroencephalogram (EEG), and ^18^F‐FDG PET/CT) were recorded systematically.

### 2.3 ^18^F‐FDG PET/CT Protocol

Brain imaging was carried out on a Discovery Elite PET/CT scanner (GE Healthcare). No sedatives were administered. After a ≥ 6 h fast, ^18^F‐FDG was injected intravenously at 3.7–5.0 MBq kg^−1^; preinjection blood glucose was required to be ≤ 8 mmol L^−1^. Patients rested quietly without external stimuli during the uptake phase. Acquisition commenced 45–60 min postinjection. Images were reconstructed with ordered‐subset expectation maximization (four iterations, eight subsets) and smoothed with a 5 mm full‐width at half‐maximum filter, using GE Advanced Workstation 4.6. Two experienced nuclear medicine physicians (Y.L.L. and A.L.), blinded to clinical data, independently performed visual interpretation of the scans.

### 2.4 Computational Analysis of PET Data

After visual inspection, each ^18^F‐FDG PET dataset was reanalyzed on a dedicated workstation with the Cortex ID suite (GE Healthcare). This software generates three‐dimensional stereotactic surface projections that complement conventional axial, sagittal, and coronal views, thereby depicting cortical tracer uptake in a surface‐rendered format. Cortex ID then performs an automated, voxel‐by‐voxel comparison with an age‐matched normative database and returns bilateral z‐scores for the following predefined regions of interest: lateral and medial prefrontal cortex, sensorimotor cortex, anterior and posterior cingulate cortex, cuneus, superior and inferior parietal cortex, lateral occipital cortex, primary visual cortex, and lateral and medial temporal cortex. The z‐score is defined as:

$$Z-\text{score}=\left[\;\text{mean subject}-\text{mean database}\right]/\mathrm{SD}\;\text{database}$$

Regional results were colorcoded on a voxel basis to illustrate the average metabolic deviation within each ROI. The z‐score threshold was higher than 2 (−1.96 based on the double tailed test), corresponding to a *p*‐value of 0.05 (double tailed)—adopted as the cut‐off for significant abnormality; positive z‐scores denoted relative hypermetabolism compared with pontine reference activity, whereas negative scores indicated hypometabolism. All computer‐generated findings were crosschecked manually to confirm anatomical accuracy and clinical plausibility.

### 2.5 Antibody Assay

Serum and CSF were screened for antineuronal antibodies directed against both surface and intracellular antigens, including NMDAR, AMPA1R, AMPA2R, LGI1, CASPR2, GABA_B‐R, IgLON5, GAD65, CV2/CRMP5, PNMA2, Ri, amphiphysin, Hu, and Yo. A fixed cell‐based assay (CBA) was performed on HEK293 cells as previously described [8]. Serum (1:10) and CSF (undiluted) were incubated with the commercial assay for 30 min, rinsed three times with buffer, and then incubated for a further 30 min with Alexa Fluor 546‐conjugated goat anti‐human immunoglobulin G (IgG) (Thermo Fisher Scientific). Fluorescent signals were evaluated by microscopy.

Indirect immunofluorescence (IIF) tissue‐based assays (TBA) were also undertaken on monkey brain sections. Sections of cerebellum and hippocampus (BioSystems S.A., 44,718) were exposed to serum (1:10) or CSF (undiluted) for 30 min, washed in PBS, and probed with mouse monoclonal antibodies specific for human IgG subclasses (IgG1, IgG2, IgG3, IgG4; SouthernBiotech). After a final PBS wash, slides were coverslipped in antifade medium and examined under a fluorescence microscope to confirm antibody binding and determine IgG subclass.

## 3 Results

### 3.1 Clinical Presentation and Immunological Profile

**FIGURE 1:**
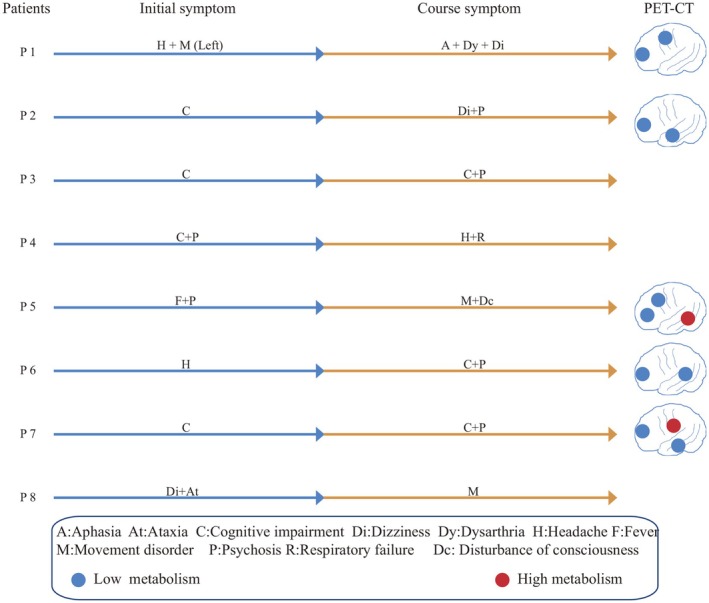
Clinical manifestations and ^18^F‐FDG PET/CT findings in individual patients. Initial presenting symptoms among the eight patients included headache, movement disorders, cognitive impairment, psychosis, ataxia, and dizziness. During the disease course, additional symptoms developed, including progressive cognitive decline, aphasia, dysarthria, persistent dizziness, psychosis, headache, respiratory failure, altered consciousness, recurrent movement disorders, and hypoxemia. The clinical features for each patient are illustrated in the figure. Patients 1, 2, 5, 6, and 7 underwent ^18^F‐fluorodeoxyglucose positron emission tomography/computed tomography (^18^F‐FDG PET/CT), which demonstrated regional hypometabolism in multiple cortical areas. Notably, Patient 5 exhibited hypermetabolism in the right temporal lobe, and Patient 7 showed increased metabolic activity in the left thalamus.

In our study of anti‐AMPAR encephalitis patients, the median age was 53 years (range 37–63). The presenting symptoms were heterogeneous: cognitive decline, headache, psychiatric disturbances, movement disorders, and ataxia. However, seizures were not observed (Figure 1). Malignancies were identified in three patients, including lung carcinoma (Patient 2), hepatocellular carcinoma (Patient 3), and thymoma (Patient 4). Patient 4 developed myasthenia gravis after antiAMPAR encephalitis. Severe disease necessitated intensive care support in Patient 4, who nonetheless achieved a good outcome. All tumor‐associated patients remained alive and functionally independent at last review (Table 1).

**TABLE 1:** Characteristics of anti‐AMPAR receptor encephalitis patients.

Patient	Age (Year)/Sex	Symptom onset until diagnosis (Weeks)	Possible predisposing factor	Immuno‐therapy or chemotherapy	Tumor state	AMPAR antibodies type	Follow up time (Months)	MRS		Prognosis CASE	Prognosis MFIS
								Hospital	Prognosis		
P1	37/F	8	—	IVMP, PLEX, IVIG, RTX	—	AMPA2‐R	19	5	4	11	19
P2	54/M	8	COVID‐19 vaccination	IVIG	Lung cancer	AMPA2‐R	25	2	0	0	1
P3	67/M	2	—	IVIG, IVMP	Liver cancer	AMPA2‐R	41	3	1	2	7
P4	36/F	4	—	IVIG, IVMP	thymoma	AMPA2‐R	48	5	0	0	0
P5	67/F	6	Fever	IVIG, IVMP	—	AMPA2‐R	77	5	1	2	5
P6	52/M	12	—	IVIG, IVMP, RTX	—	AMPA2‐R	15	2	Death	Death	Death
P7	57/M	24	—	IVIG	—	AMPA1‐R	12	1	1	2	4
P8	51/F	4	—	IVMP, MMF	—	AMPA2‐R	NA	2	NA	NA	NA

*Note:* Patient 6 (P6) died of suspected meninges carcinoma in other hospital.

Abbreviations: AMPA, anti‐alpha‐amino‐3‐hydroxy‐5‐methyl‐4‐isoxazole propionic acid; CASE, clinical assessment scale for autoimmune encephalitis; F, Female; IVIG, intravenous immunoglobulin; IVMP, intravenous methylprednisolone; M, male; MFIS, modified fatigue impact scale; MMF, mycophenolate mofetil; MRS, Modified Rankin Scale; NA, not available; P, patient; PLEX, plasma exchange; RTX, rituximab.

**FIGURE 2:**
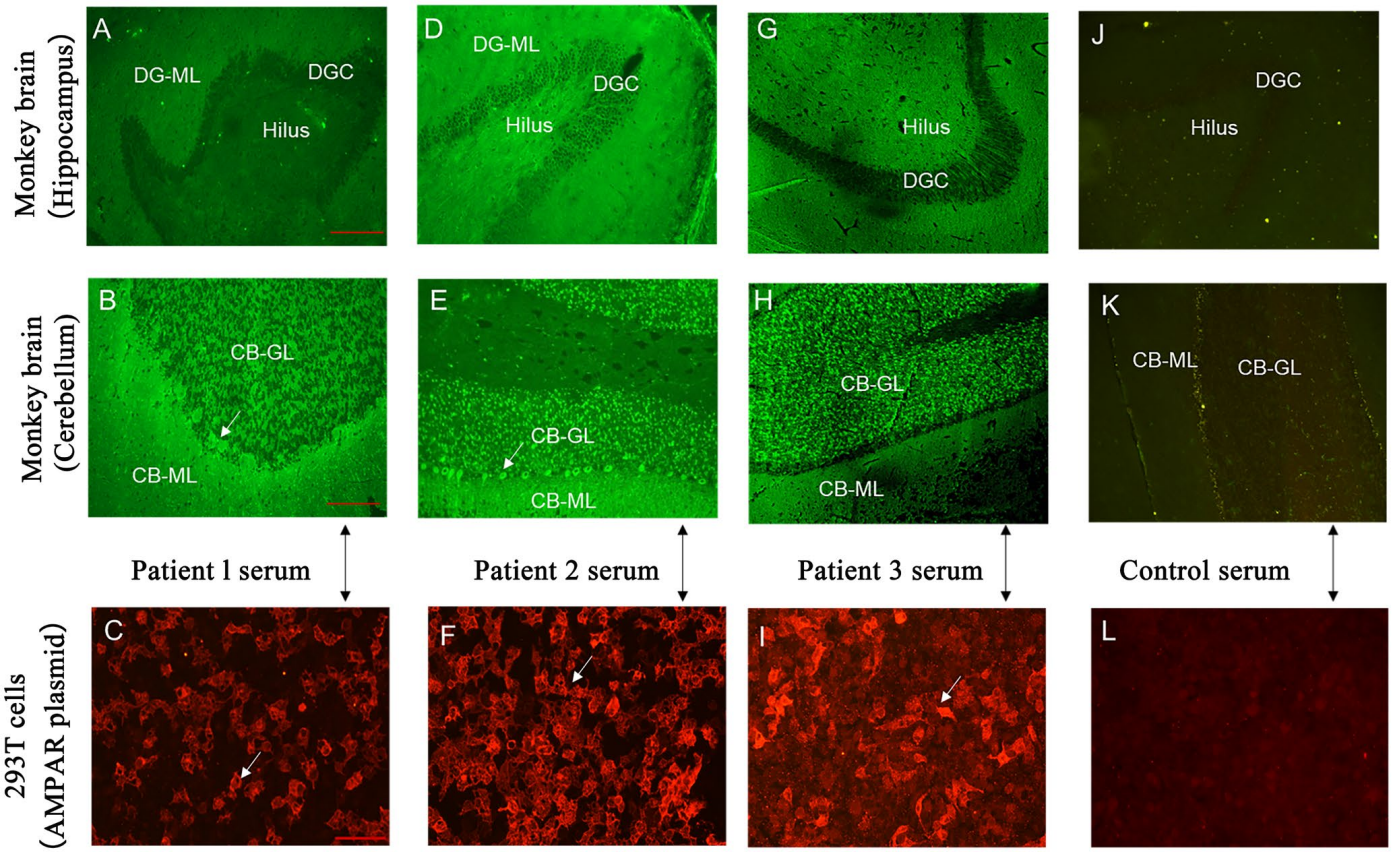
Anti‐AMPA receptor (AMPAR) antibodies were identified using indirect immunofluorescence on tissue‐based assays (IIF‐TBA) with monkey brain tissue. Antibody binding to the neuropil of the hippocampus (A, D, G, J) and the molecular layer of the cerebellum (B, E, H, K) was observed in samples from three patients. White arrows indicate antibody‐positive cells, while no signal was detected in control tissues (J, K). Higher magnification images (D and G, 40×; scale bar: 100 μm) provide enhanced visualization of immunoreactive cells. IgG subclass analysis from serum samples (C, F, I) revealed that the anti‐AMPAR antibodies belonged to the IgG1 subclass (scale bar: 20 μm; white arrows). Strong immunoreactivity was observed on AMPAR‐transfected 293 T cells (C, F, I, L), while no signal was detected in control‐transfected cells (L), confirming assay specificity. Within the cerebellar tissue, the molecular layer (CB‐ML), dentate gyrus molecular layer (DG‐ML), and cerebellar granular layer (CB‐GL) exhibited intense and homogeneous antibody binding, with the CB‐GL showing mild‐to‐moderate punctate staining (B, E, H). The figure also includes comparative immunofluorescence findings from healthy monkey brain tissues and serum controls, as well as immunohistochemical (IHC) detection.

Anti‐AMPA2R antibodies were detected in serum and/or CSF in seven patients, whereas one patient carried anti‐AMPA1R antibodies. In three cases, tissue‐based immunofluorescence confirmed IgG1 subclass anti‐AMPAR antibodies with strong reactivity in cerebellar and hippocampal neurons (Figure 2). Collectively, these findings indicate that anti‐AMPAR encephalitis in this series was dominated by IgG1 antiAMPA2R antibodies and accompanied by modest inflammatory CSF changes, supporting a robust humoral immune response directed against limbic and cerebellar structures. In addition to anti‐AMPAR antibodies, some patients also have other autoimmune antibodies, including antinuclear antibodies, anti‐amphiphysin, and anti‐CV2 antibodies (Table 2).

**TABLE 2:** Examination results of anti‐AMPAR receptor encephalitis patients.

Patient	Interval between symptom onset and PET imaging (weeks)	MRI		EEG	CSF		OB	Other autoimmune encephalitis antibodies	Positive of systemic autoimmune antibodies	Increased cell factor	
		Long T1 and T2	Unremarkable		WBC	Protein				Serum	CSF
P1	10	Left temporal lobe, Frontal cortex and subcortical, Superior cerebellar vermis;		NA	4/ul	82.7 mg/dL	NA	—		TNF‐α, IL‐6, IL‐1β	NA
P2	9		√	NA	8/ul	26.64 mg/dL	+	Amphiphysin, Anti‐CV2		—	
P3	3	Bilateral hippocampus		Slow waves in the temporal and occipital	64/ul	77.6 mg/dL	+	—		IL‐6	IL‐6, IL‐8, IL‐1β
P4	6	Bilateral temporal lobes		Slow activity	82/ul	41.5 mg/dL	+	—		—	—
P5	7	Bilateral frontal subcortex, Lateral ventricle and temporal lobes		NA	7/ul	22.57 mg/dL	+		ANA (1;1000)	NA	NA
P6	13	Left thalamus and insular lobe		NA	25/ul	346.55 mg/dL	—	—			IL‐6, IL‐8
P7	24		√	—	1/ul	35.77 mg/dL	+	—		IL‐1β	TNF‐α, IL‐6
P8	5		√	—	4/ul	33 mg/dL	NA		ANA (1;80)	NA	NA

*Note:* Serum and CSF factor standard value, CSF‐WBC (0–8/ul), CSF‐Protein (15‐45 mg/dL), TNF‐α (0.00–8.10 pg/mL), IL‐6 (0.00–3.40 pg/mL), IL‐1β(0.00–5.00 pg/mL).

Abbreviations: −, negative; +, positive; ANA, antinuclear antibody; CSF, cerebrospinal fluid; EEG, electroencephalogram; F, Female; IL, interleukin; MRI, magnetic resonance imaging; NA, not available; OB, oligoclonal bands; P, patient; PET, positron emission tomography; TNF, tumor necrosis factor; WBC, white blood cells.

### 3.2 Neuroimaging Patterns and Other Ancillary Findings

**FIGURE 3:**
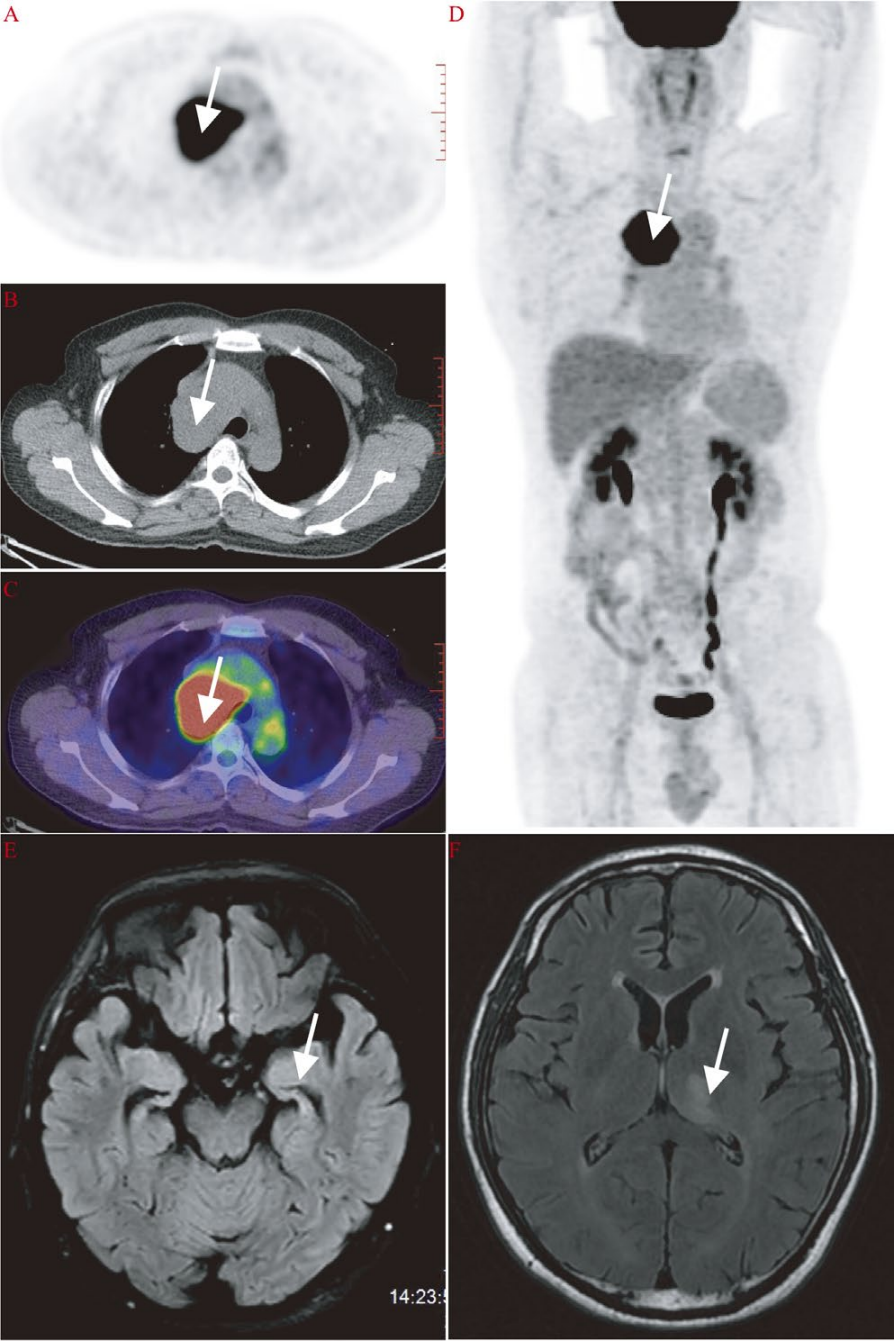
Representative MRI and ^18^F‐FDG PET/CT findings in patients with anti‐AMPAR encephalitis. Whole‐body ^18^F‐FDG PET/CT imaging of Patient 2 revealed a hypermetabolic pulmonary lesion consistent with lung cancer, indicated by white arrows (A–D). Brain MRI of Patient 3 demonstrated bilateral hyperintense signals in the medial temporal lobes on T2‐FLAIR sequences, predominantly affecting the hippocampi (E). In Patient 7, T2‐FLAIR imaging showed a focal hyperintensity in the left thalamus (F), suggestive of localized inflammation or metabolic dysfunction.

**FIGURE 4:**
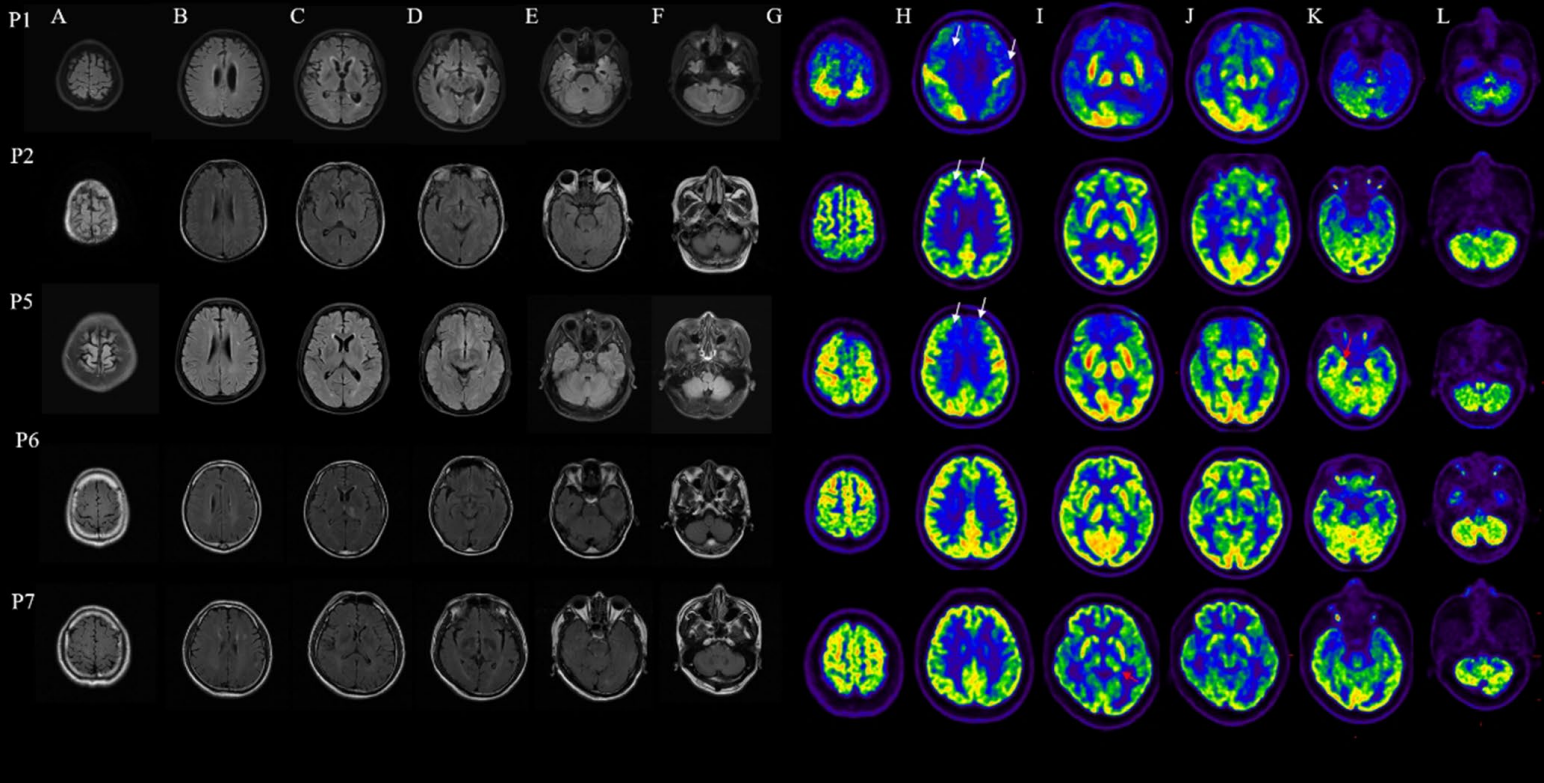
Brain MRI and ^18^F‐FDG PET/CT imaging in patients with anti‐AMPAR encephalitis. The MRI images of the brain are shown for Patients 1, 2, 5, 6, and 7 (A–F). Axial slices of ^18^F‐FDG PET/CT images from the vertex to the skull base are presented in panels (G–L). White arrows indicate regions of cortical hypometabolism, predominantly in the prefrontal cortex. In contrast, red arrows highlight areas of hypermetabolism. Patients 1, 2, and 5 exhibited marked prefrontal hypermetabolism (P1‐H, P2‐H, P5‐ H). Additionally, Patient 5 demonstrated hypermetabolism in the right temporal lobe (P5‐K), and Patient 7 showed increased metabolic activity in the left thalamus (P7‐K), consistent with focal neural activation or inflammation. ^18^F‐FDG PET/CT shows greater sensitivity than MRI.

**FIGURE 5:**
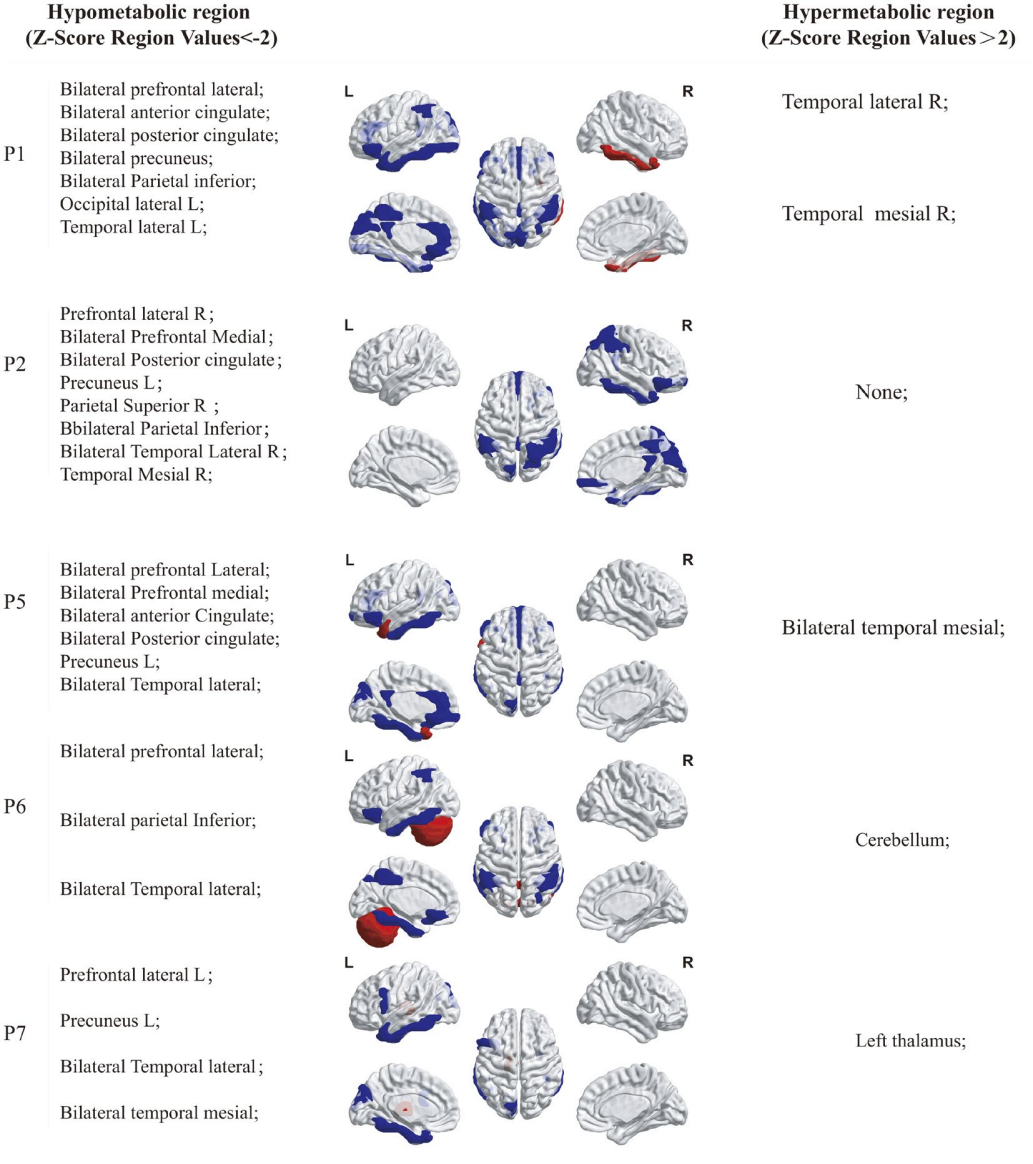
Hypometabolic and Hypermetabolic Regions in Five Patients Quantified by Z‐Score. Hypometabolic region (Z‐Score Region Values< −2) and Hypermetabolic region (Z‐Score Region Values > 2) are shown in Figure. The blue indicates regions of hypometabolism and the red indicates regions of hypermetabolism. All five subjects displayed prefrontal hypometabolism; three (P1, P2, P5) also showed posteriorcingulate hypometabolism. P: Patient; L: Lift; R: Right.

Five patients underwent ^18^F‐FDG PET/CT, all demonstrating cortical or subcortical dysmetabolism. Marked hypometabolism (z‐score < −2) in the prefrontal cortex and posterior cingulate gyrus was a consistent feature. Focal hypermetabolism was also recorded in the right temporal lobe (Patient 5) and left thalamus (Patient 7). Temporal lobe hypermetabolism in Patient 1 and cerebellar hypermetabolism in Patient 6 further underscored the heterogeneity of metabolic change. Brain MRI abnormalities were detected in 5 of 8 patients (62.5%). Bilateral hippocampal T2/FLAIR hyperintensities were present in three cases. Representative MRI and ^18^F‐FDG PET/CT findings in patients with lung cancer are shown in Figure 3. The patients' MRI findings do not completely align with the metabolic abnormalities in ^18^F‐FDG PET/CT, and ^18^F‐FDG PET/CT shows greater sensitivity (Figure 4; Figure 5). Routine EEG in Patients 3 and 4 revealed diffuse slow‐wave activity over temporal and occipital regions but no epileptiform discharges; clinically, seizures were absent throughout the series (Table 2).

### 3.3 Treatment Strategies and Clinical Outcomes

All eight patients received guideline‐based first‐line immunotherapy for autoimmune encephalitis [9], namely high‐dose intravenous methylprednisolone (IVMP), intravenous immunoglobulin (IVIG), and/or plasma exchange (PLEX). Second‐line immunomodulation was required in three cases, including rituximab (RTX) and mycophenolate mofetil. The patients had a good response to immunotherapy.

## 4 Discussion

The metabolic status of brain tissue measured by ^18^F‐FDG PET/CT may provide an objective correlate of extracranial vascular hemodynamics. Regional metabolic heterogeneity across cerebral lobes has been noted in anti‐AMPAR encephalitis, yet the precise contribution of PET‐derived metabolic data to interpreting cerebral blood flow and functional neurologic impairment remains uncertain. In our cohort, symptom expression was heterogeneous but most often limbic, cognitive decline and abnormal mental behavior predominated, suggesting that regional receptor vulnerability shapes the clinical picture. Most patients eventually achieved good functional recovery, with only mild residual memory loss or fatigue and little fixed disability. The broad symptom spectrum (cognitive, behavioral, and motor) reported here and elsewhere is consistent with the anatomic distribution of AMPAR subunits in the brain [9].

AMPAR is an ionotropic glutamate receptor expressed at high density in both gray and white matter. Antibody‐dependent internalization or removal of synaptic AMPARs perturbs ion flux and attenuates excitatory signaling [10, 11, 12]. Exposure of neurons to IgG from AMPAR‐positive patients reduces the peak amplitude of miniature excitatory postsynaptic currents (mEPSCs) and lengthens inter‐event intervals; the resulting loss of AMPAR‐containing synapses increases the proportion of “silent” synapses in which quantal glutamate release no longer elicits measurable mEPSCs. The precise synapse‐specific mechanisms that drive these antibody effects remain unclear [13]. Anti‐AMPAR antibodies therefore serve as useful markers for distinguishing suspected autoimmune encephalitis from healthy controls and from other neurologic disorders [10, 14, 15]. Additional neural or systemic autoantibodies have been described in some patients, a pattern we also observed. In contrast to IgLON5 disease, typically dominated by IgG4 with variable IgG1 components [16], our series demonstrated a predominantly IgG1 anti‐AMPAR response, consistent with prior reports [17]. IIF‐TBA highlighted strong staining of monkey cerebellar and hippocampal tissue by patient samples, underscoring the anatomic targets of the immune response. AMPAR antibodies chiefly recognize GluA1 and GluA2 epitopes, with particular emphasis on GluA2. Our findings, along with others [18, 19, 20], hence, indicate that anti‐AMPA2 receptor encephalitis is more common than anti‐AMPA1 disease.

Our neuroimaging data align with immunologic observations. Patients 2 and 6 lacked limbic hypermetabolism, possibly reflecting low inflammatory burden and imaging performed during clinical improvement. In contrast, five patients showed focal or regional hypermetabolism, which may relate to delayed diagnosis and scanning during active inflammation. Prefrontal hypometabolism emerged as a recurring pattern and may contribute to the prominent neuropsychiatric manifestations observed. More broadly, ^18^F‐FDG PET/CT dysmetabolism spanning cortical and subcortical regions paralleled key clinical features of anti‐AMPAR encephalitis and supports a link to disrupted glutamatergic signaling.

Due to the limited number of cases and absence of control, more detailed work should be further implemented. In our study, we could not determine whether concomitant tumors, additional autoantibodies at onset, or specific immunosuppressive regimens influence prognosis. One patient developed anti‐AMPAR encephalitis after thymectomy and was later diagnosed with myasthenia gravis, underscoring the complex autoimmune networks associated with thymic disease [21]. Reported malignancy associations include lung cancer, breast cancer, and thymoma [22, 23]. Given the predominance of IgG1, and the likely B cell‐mediated humoral mechanism, therapies that reduce or neutralize circulating antibodies (PLEX, IVIG) or deplete B cells are mechanistically plausible and were associated with clinical improvement in most of our patients. Furthermore, effective immunotherapy has been associated with the alleviation of clinical symptoms.

Comparison with anti‐NMDAR encephalitis is informative. That disorder exhibits increased cerebral blood flow (CBF) in the putamen and amygdala with reduced flow in the right anterior cingulate gyrus, findings that mirror its characteristic ^18^F‐FDG PET/CT topography [24]. Whether a comparable perfusion–metabolism relationship exists in anti‐AMPAR disease remains unknown. Prospective multimodal studies incorporating quantitative CBF imaging, metabolic mapping, and longitudinal clinical assessment are needed to clarify cerebrovascular contributions to pathogenesis and recovery in anti‐AMPAR encephalitis.

## 5 Conclusions

^18^F‐FDG PET/CT highlights metabolic alterations that may mirror regional cerebral blood flow in anti‐AMPAR encephalitis. Although clinical presentations are varied, they most commonly align with a limbic encephalitis phenotype, and neither relapses nor epileptic seizures occurred during follow‐up in this series. Imaging consistently demonstrated widespread lobar hypometabolism, with the prefrontal cortex and posterior cingulate emerging as probable metabolic hallmarks of the disease. Focal hypermetabolism in the basal ganglia or temporal lobes appeared only intermittently. These findings probably underscore the metabolic imaging for recognizing anti‐AMPAR encephalitis as one of its diverse clinical features.

## Author Contributions

W.S., L.A., and W.M. conceived the research; H.C., L.L., P.Z., and X.X. designed the research and wrote the manuscript; Y.M., H.Z., Y.X., Y.X.u., M.Z., and B.L. collected clinical specimens; W.D., L.L., G.C., and W.X. performed the data processing; all authors reviewed and approved the final manuscript.

## Ethics Statement

This study was performed with the approval of the Ethics Committee of the Beijing Tiantan Hospital, Capital Medical University (Ethics Committee document number: KY 2023–064‐01). The Declaration of Helsinki was obeyed as a statement of ethical principles for this research.

## Consent

Participants' informed consent was obtained included in this study.

## Conflicts of Interest

The authors declare no conflicts of interest.

## Data Availability

The data that support the findings of this study are available from the corresponding author upon reasonable request.
